# Parent-infant interactions in families with women diagnosed with postnatal depression: a longitudinal study on the effects of a psychodynamic treatment

**DOI:** 10.3389/fpsyg.2015.01210

**Published:** 2015-08-11

**Authors:** Renata Tambelli, Luca Cerniglia, Silvia Cimino, Giulia Ballarotto

**Affiliations:** ^1^Department of Dynamic and Clinical Psychology, Sapienza University of RomeItaly; ^2^Department of Psychology, International Telematic University UNINETTUNORome, Italy

**Keywords:** parent-infant interactions, post-partum depression, fathers, psychodynamic treatment, temperament

## Abstract

**Background:** Several studies have shown a connection between mothers with postnatal depression (PND) and emotional-behavioral problems in their children. Mothers’ psychopathology may impair interactional patterns with children and these outcomes can be influenced by father’s psychopathological symptoms. The primary aim of the study was to assess over time parent-infant interaction in families where mothers have experienced PND and have received psychological treatment during the child’s first year of life considering the severity of parents’ psychopathological symptoms and children’s temperament.

**Methods:** Three groups of families were involved: families with mothers with PND wherein both parents followed a psychological treatment (TxMF); families with mothers affected by PND wherein only the mother followed the treatment (TxM) and control families wherein the mothers did not have a psychopathological diagnosis and did not receive any treatment (Con). The families were assessed at two time points through Symptom Check-List-90-Revised (SCL-90-R), Questionari Italiani Temperamento (QUIT) and the video-recorded procedure observing mealtime Scala di Valutazione Interazioni Alimentari (SVIA).

**Results:** Parents in the TxMF group had significantly lower SVIA scores (i.e., less maladaptive) at T2. TxMF group scored lower at T2 at SCL-90-R, whereas TxM showed no significant differences between T1 and T2. Involvement of fathers in the treatment was important to improve the psychopathological symptoms of both parents and the quality of interactions with their children.

## Introduction

Postnatal depression (PND) and its possible consequences for both the child’s emotional adaptive functioning and the relational patterns in mother–child dyads have been examined thoroughly in recent international studies (Korja et al., 2008). Epidemiological data show that ∼13% of women suffer from symptoms of PND which do not improve or may worsen in the first few weeks after giving birth; these symptoms can also appear in the period immediately before the birth (Séjourné et al., 2012), as specified in the recently updated Diagnostic and Statistical Manual of Mental Disorders (DSM-5, American Psychiatric Association [APA], 2013). Research in this field has, thus far, focused mainly on the severity of mother’s psycho-pathological symptoms associated with a PND diagnosis (Sutter-Dallay et al., 2011) and only recently has the work been expanded to consider possible paternal contributions to risk and protection factors (Paulson and Bazemore, 2010; Cimino et al., 2014).

The large amount of research on mothers to date has highlighted the relationship between a PND diagnosis and emotional and behavioral problems in children during their development, especially with respect to externalizing syndromes (Madigan et al., 2007). Cicchetti and Rogosch (1996), however, have proposed a multifinality model that suggests that maternal depression can generate maladaptive outcomes in children, manifested as externalizing as well as internalizing syndromes, and can generally be associated with altered emotional functioning in children. Postnatally depressed mothers who show withdrawn interaction with their children seem more likely to have offspring manifesting internalizing functioning characterized by withdrawal, anxiety and somatic complains, while PND mothers’ intrusive behaviors seem to foster externalizing problems such as aggressive behaviors in their children (Goodman et al., 2011, 2015).

Coherently with the above research, specific characteristics of mother-infants interactions have been studied and there is a consensus in the literature that early exchanges between mothers with PND and their infants are characterized by reduced face-to-face interaction, reduced frequency of positive facial expressions and of fluid and contingent turn-taking, such as during meals (Ammaniti et al., 2012). The quality of feeding interactions between parents and their children during the first years of life have been defined as proxy for more global quality of parent-infant interactions since these exchanges constitute fundamental relational experiences having weight on adaptive or maladaptive child’s development (Field et al., 2006). In an intersubjective perspective, the quality of parent-infant interactions during feeding or play not only have effect on children’s development and psychological wellness, but it also depend on the capacity of the parent of adapting and modulating the specific characteristics of the child, such as his/her temperament, behavior and general psychological functioning (Stern, 1995; Cerniglia et al., 2014a). The characteristics of the interaction between mothers with PND and their children often correlate with the mother’s difficulties with establishing a syntonic relationship with her child, a circumstance which can contribute to the development of insecure attachment patterns (Madigan et al., 2007).

On the other hand, several authors have shown the importance of considering additional risk factors that may lead to emotional problems in children whose mothers have PND, such as the presence and especially the severity of maternal psychopathological symptoms associated to PND. Besides, it has been shown that maladaptive outcomes in children of mothers with PND may be linked to father-infant interactions characterized by asynchrony, scarce involvement, and an absence of sharing positive affective bonds (Dietz et al., 2009; Beebe et al., 2012). The international literature suggests that the emotional-adaptive difficulties in parental interactions (e.g., during play or mealtime) with children whose mothers have received a psychiatric diagnosis can be influenced by the severity of their father’s psychopathological symptoms (Pinquart and Teubert, 2012). Hence, it has been underscored how useful it would be to consider the presence of psychopathological symptoms in fathers, whenever these cannot be classified within a specific nosographic category (Cimino et al., 2013). It has also been emphasized that temperamental and individual factors in children (as perceived by the parents) can interact with parents’ emotional problems and contribute to the onset of psychological difficulties in the children and to the development of difficulties in parent–child dyadic relationships with both parents as well as the triadic one (Klein et al., 2009). In this paper we refer to Rothbart et al. (2000) definition of temperament as biologically based and relatively stable over time but modifiable by development and experiences in the environment.

With regards to the treatment of maternal PND, Murray et al. (2010) studied the improvements in mother-infant relationships and infant developmental outcomes subsequent to different intervention approaches (Cognitive Behavioral Therapy, Counseling and Psychoanalytic Therapy). All the treatments in this study produced short-term benefits on mothers’ depression, but there was scarce evidence of benefits to maternal mood at follow-up. Interestingly, this study also demonstrated that although psychological treatments were effective in treating maternal depression, no positive effect was found in terms of observed mother-infant interactions, infant negative emotionality, and infant attachment security (Cooper et al., 2010).

Field (2010) suggested that such treatments should involve not only mothers with PND, but rather the whole family group. In fact, it has been proposed that not only do the mother’s psychopathological symptoms improve with whole family treatment, but also that reduction of the father’s psychopathological risk (which may intensify during the postpartum period) can improve the children’s psychological wellbeing and the quality of the dyadic mother–child exchanges (Goodman, 2008; Edmondson et al., 2010; Lucarelli et al., 2013). Although recent literature and research have convincingly demonstrated that considering paternal psychopathological risk and its effects on children development in families with mentally ill mothers can guarantee better results on the family psychological wellness, several mental health services continue to offer treatment plans addressing mothers only, excluding fathers from intervention polices (Ramchandani et al., 2013; Werner et al., 2015).

## Materials and Methods

The general objective of this work was to assess the quality of mother-infant and father-infant interactions in families in which the mother has PND and has followed a psychodynamic treatment during the child’s first year of life. The following three participant groups were compared: families with maternal PND wherein the mother and father followed a psychodynamic treatment during the child’s first year of life (TxMF); families with maternal PND wherein only the mother followed a psychodynamic treatment during the child’s first year of life (TxM); and control families wherein the mothers do not have a psychopathological diagnosis and parents did not receive any kind of treatment (Con). (See **Table 1** for demographic characteristics of the sample).

**Table 1:** Demographic characteristics of participants by group.

Group	*N*	Gender	Mean age ± SD			
			Mothers, y	Fathers, y	Infants at T1, mos.	Infants at T2, mos.
TxMF	22	11 M, 11 F	31.9 ± 1.8	35.5 ± 2.2	3.1 ± 0.3	12.2 ± 0.6
TxM	22	11 M, 11 F	31.7 ± 1.9	36.2 ± 1.8	3.2 ± 0.2	11.9 ± 0.4
Con	22	11 M, 11 F	31.2 ± 2.0	38.1 ± 3.2	3.4 ± 0.3	12.1 ± 0.5

The primary objective of this study was:

(a) To assess changes or stability in the quality of relational mother-infant and father-infant exchanges during meals in the three groups;

The secondary objectives of the study were:

(b) To assess changes or stability in the severity of psychopathological risk (in terms of global functioning) of mothers and fathers in the three groups;
(c) To compare longitudinally the temperamental characteristics of children in the three groups as perceived by their parents.

### Subjects and Procedure

Over a 1-year period, 293 families expecting a baby addressed a network of public consultants in Central Italy following a program to accompany them to the child’s birth and into parenthood. To establish a baseline of parental psychopathological risk before birth, we administered the Symptom Checklist-revised (SCL-90-R) to all parents at the sixth month of pregnancy. For this study, we excluded families with other children (*N* = 104). After the child’s birth, we excluded families if the mother and father were not handling personally the child’s care and nutrition (for example delegating the child’s feeding to grandparents because mothers and fathers are at work during the day; *N* = 31). In the remaining sample group (*N* = 158), 87 mothers were diagnosed with PND without comorbidity by psychiatrists from the various consultant offices, according to the DSM-5 criteria (American Psychiatric Association [APA], 2013) within the first 4 weeks of the child’s life, 49 mothers received a different diagnoses (*N* = 11 anxiety disorder; *N* = 8 borderline personality disorder; *N* = 16 PND with a comorbid anxiety disorder, and *N* = 14 nutritional disorder) and were suggested to follow treatments that were not included in this study, and 22 mothers received no diagnosis [and constituted Control Group (Con)]. Among the families with mothers with PND a cohort of 52 families elected to accept the assessment and treatment plan, while 35 families refused to participate in the study. Of the 52 families that commenced with the intervention, eight did not adhere to the treatment (*N* = 2 moved to another city after two treatment sessions; *N* = 6 families did not keep their appointments after the first session), yielding a drop-out of 15.3%. Only those families who followed the program continuously to its conclusion were included in the data analysis (*N* = 44). The families were randomly assigned (by computer software which was blind to socio-demographical and psychological characteristics of the global sample) to different treatment plans: *N* = 22 families were assigned to an intervention involving both parents (TxMF), and *N* = 22 involving only the mother (TxM). None of the parents in Group TxMF, Group TxM, and Control Group (Con) exceeded cut-offs for the Italian population at SCL-90-R (Prunas et al., 2012) administered at sixth month of pregnancy of the mother.

The network of public consultants offered the families of Group (TxMF) and Group (TxM) a supportive, relationship-based, parent-infant intervention, which was developmentally based and infant-oriented, promoting positive parent-infant interactions. The intervention was drug-free and included 15 meetings (two sessions a month; 1 h per session). The same treatment technique was proposed to TxMF and TxF Groups but, when both parents were involved, part of the session was dedicated to confronting mothers and fathers’ characteristics in interacting with their child. The clinical équipe was composed of five psychologists within the public health care system specifically trained for treatment techniques described by Cramer et al. (1990), Stern (1995) in which an understanding of the parents’ representation of her infant and her relationship with their infant was promoted by exploring aspects of the parents’ early experiences with their own families. The health care public services where the sample was recruited chose to offer this specific treatment to their patients because a robust literature demonstrated its high efficacy on families with women with PND (Nylen et al., 2006; Cooper et al., 2010).

The groups were evaluated through the tools described below at two time points with an inter-evaluation interval of ∼9 months. The first time point (T1) was when the children were 3 months old (preceding the start of intervention in TxMF and TxM) and the second time point (T2) was when they were 12 months old (immediately after treatment conclusion).

The research described here was approved by the Ethical Committee of the Psychology Faculty at Sapienza, University of Rome, before the start of the study and in accordance with the Declaration of Helsinki. Written informed consent was obtained from each of the study participants.

### Tools

All parents were administered both at T1 and T2 through the *90-item Symptom Checklist-revised* (SCL-90-R; Derogatis et al., 1973) and an Italian questionnaire on temperament [*Questionari italiani del temperamento* (QUIT); Axia, 2002] independently. For the present study we considered only the Global Severity Index (GSI) as an index of general psychopathology, since recent international research has doubted the validity of the SCL-90-R subscales and recommend the use of GSI (Müller et al., 2011). Also, mother-infant and father-infant nutrition interactions were video-recorded and evaluated with the “*Scala di Valutazione dell’Interazione Alimentare*” (SVIA; Lucarelli et al., 2002).

#### Symptom Check-List-90-Revised

The SCL-90-R is a self-report questionnaire that gives a standardized measure of the current psychological and/or psychopathological status of a subject that can be applied to normal or psychiatric adult and adolescent populations. It provides a wide range of information on the current subjective experience of psychological wellbeing and distress, and serves as a screening tool in both clinical and research settings. The scores obtained are interpreted based on nine primary dimensions: (1) *somatization*, (2) *obsession compulsion*, (3) *interpersonal sensitivity*, (4) *depression*, (5) *anxiety*, (6) *hostility*, (7) *phobic anxiety*, (8) *paranoid ideation*, and (9) *psychoticism.* It includes a GSI that is used to determine severity and degree of psychological distress with respect to the nine primary dimensions measured. Prunas et al. (2012) demonstrated satisfactory internal consistency of the Italian version of the SCL-90-R in adolescents and adults (α coefficient, 0.70–0.96) with a clinical cut-off score ≥1 in GSI indicating psychopathological risk (Prunas et al., 2012).

#### Questionari Italiani Temperamento

The QUIT is a 60-item parent questionnaire that was validated in an Italian sample across four age bands: 1–12 months, 13–36 months, 3–6 years, and 7–11 years. It investigates six dimensions referring to the child’s temperament as perceived by the parents: (1) *level of motor activity*, (2) *attention capacity*, (3) *inhibition to novelty*, (4) *social orientation* (readiness for socialization), (5) *positive emotionality*, and (6) *negative emotionality*. The questionnaire shows good internal consistency for the 1–12-month age band (Cronbach’s α, 0.59–0.71; Axia, 2002) and in the present study mothers’ and fathers’ independent questionnaire responses have been shown to have good agreement (Pearson’s *r* = 0.87) in all three Groups (TxMF, TxM, Con).

#### Scala di Valutazione Interazioni Alimentari

The SVIA is the Italian adaptation of the Feeding Scale (Chatoor et al., 1997) that can be applied to children that are 12–36 months old. It measures interactive behaviors and identifies normal and/or risky relational modes between a parent and child during feeding exchanges (Lucarelli et al., 2002). Parent-infant interactions during feeding are recorded for at least 20 min, and then a wide range of interactive mother-infant behaviors are coded and evaluated.

The SVIA consists of 41 items distributed among four subscales: (1) *Parent’s affective states* (index of the parent’s affective states); (2) *Interactive conflict* (index of interactions characterized by conflictual, non-collaborative, and non-empathetic communication); (3) *Food refusal behavior* (habits associated with challenged status regulation during meals and with limited food consumption); and (4) *Dyad’s affective state* (index of the extent to which the infant’s feeding patterns are, or are not, the result of an interactive regulation to which both partners contribute). The scores, measured on 4-point a Likert Scale ranging from 0 to 3 (none, a little, quite a bit, a lot), for each subscale were compared with standard values from the Italian standardized sample.

Inter-evaluator agreement for SVIA items is generally good to excellent (Pearson *r* values, 0.7–1.0 for group of 182 normal infants and 0.9–1.0 for a group of 182 infants with nutritional disorders). And the instrument shows good reliability, in terms of internal consistency (Cronbach’s α, 0.79–0.96).

### Data Analysis

Scores were compared between the groups with analyses of variance (ANOVAs) for repeated measures. The time elapsed between the two administrations (from T1 to T2) was treated as a within-subject factor and the belonging to a research groups was treated as a between-subjects factor. Bonferroni’s and Scheffè’s *post hoc* tests were applied. The calculated *p* values are reported with their respective F statistics and degrees of freedom (df), with values <0.05 being accepted as significant. Mean values are reported with SDs. A power analysis was conducted accordingly to Cohen’s (2013) suggestions, *α* was set at 0.05 and a power of 0.832 was obtained with a large effect size of (*f*^2^ = 0.40).

All statistical analyses were performed by SPSS version 18.0.

## Results

Most of the families recruited for the study (88%) had a middle socio-economic status (Bornstein and Bradley, 2014; Hollingshed, unpublished), and a large majority (92%) were intact family groups in which the child was the firstborn for both parents. The families were 91% Caucasian and 71% relied on more than one income. All of the babies were breastfed and formula fed (mixed fed) and all of the fathers took part in the children’s caretaking and feeding routine.

### Longitudinal Assessment of the Quality of Relational Mealtime Exchanges by Group

An ANOVA revealed main effects of group (all *p* < 0.001) and time point (all *p* < 0.001) on all four SVIA subscale scores for mothers in the TxMF group. Bonferroni’s *post hoc* tests demonstrated that mothers in the TxMF group had significantly lower scores (i.e., less maladaptive) at T2 versus T1 for all four subscales: *mother’s affective state*; *interactive conflict*; *food refusal*; *dyad’s affective state*. The subscale scores of the mothers in the TxM and Con groups did not differ significantly between T1 and T2. The mothers’ average scores for each SVIA subscale at T1 and T2, F and η^2^ values are reported in **Table 2**.

**Table 2:** Mean Scala di Valutazione Interazioni Alimentari (SVIA) subscale scores ± SD, *F* and η^2^ at T1 and T2 by group for mothers.

SVIA	TxMF				TxM				Con			
	T1	T2	Fisher *F* test	η^2^	T1	T2	Fisher *F* test	η^2^	T1	T2	Fisher *F* test	η^2^
MAS	24.07 ± 2.18	15.13 ± 1.86	*F*_1,39_ = 220.81^∗∗^	0.42	23.87 ± 2.39	24.09 ± 2.02	*F*_1,39_ = 60.45	0.03	14.6 ± 1.59	15.11 ± 1.85	*F*_1,39_ = 80.98	0.07
IC	22.47 ± 1.83	7.57 ± 1.65^∗∗^	*F*_1,39_ = 791.79^∗∗^	0.35	22.22 ± 2.00	21.7 ± 2.05	*F*_1,39_ = 146.56	0.05	7.21 ± 1.34	7.35 ± 1.54	*F*_1,39_ = 166.71	0.07
FR	12.77 ± 1.61	7.09 ± 1.19^∗∗^	*F*_1,39_ = 171.12^∗∗^	0.48	12.59 ± 1.65	13.00 ± 1.19	*F*_1,39_ = 46.87	0.08	6.86 ± 1.36	7.07 ± 1.17	*F*_1,39_ = 61.36	0.08
DAS	15.17 ± 1.51	3.99 ± 0.68^∗∗^	*F*_1,39_ = 920.04^∗∗^	0.46	15.23 ± 1.43	14.93 ± 1.76	*F*_1,39_ = 341.23	0.02	3.84 ± 0.62	3.90 ± 0.57	*F*_1,39_ = 189.53	0.05

*^∗∗^p < 0.001. MAS, mother’s affective state; IC, interactive conflict; FR, food refusal; DAS, dyad affective state.*

An ANOVA also revealed main effects of group (all *p* < 0.001) and time point (all *p* < 0.001) on fathers’ scores for all four SVIA subscale scores. Similar to our findings with the mothers, we observed that fathers in the TxMF group had significantly lower scores (i.e., less maladaptive) at T2 versus T1 for all four subscales: *father’s affective state*; *interactive conflict*; *food refusal*; *dyad’s affective state*. The subscale scores of the fathers in the TxM and Con groups did not differ significantly between T1 and T2. The fathers’ mean scores for each SVIA domain at T1 and T2, F and η^2^ values are shown in **Table 3**.

**Table 3:** Mean SVIA subscale scores ±SD, *F* and η^2^ at T1 and T2 by group for fathers.

**SVIA**	**TxMF**				**TxM**				**Con**			
	**T1**	**T2**	**Fisher *F* test**	**η^**2**^**	**T1**	**T2**	**Fisher *F* test**	**η^**2**^**	**T1**	**T2**	**Fisher *F* test**	**η^**2**^**
FAS	23.46 ± 2.66	14.89 ± 2.00^∗∗^	*F*_1,39_ = 148.16^∗∗^	0.61	22.04 ± 2.69	22.41 ± 2.82	*F*_1,39_ = 71.32	0.03	15.21 ± 1.82	15.10 ± 2.10	*F*_1,39_ = 86.78	0.04
IC	21.48 ± 2.67	7.77 ± 1.60^∗∗^	*F*_1,39_ = 360.60^∗∗^	0.45	20.67 ± 2.50	20.82 ± 2.30	*F*_1,39_ = 189.73	0.05	7.42 ± 1.51	7.75 ± 1.45	*F*_1,39_ = 172.13	0.06
FR	12.62 ± 1.76	6.88 ± 1.18^∗∗^	*F*_1,39_ = 176.65^∗∗^	0.55	12.10 ± 1.51	12.23 ± 1.54	*F*_1,39_ = 89.12	0.06	7.08 ± 1.18	6.91 ± 1.18	*F*_1,39_ = 95.49	0.07
DAS	14.83 ± 1.96	4.04 ± 0.60^∗∗^	F_1,39_ = 532.58^∗∗^	0.38	13.37 ± 1.63	13.85 ± 1.63	*F*_1,39_ = 223.41	0.02	3.88 ± 0.59	4.02 ± 0.55	*F*_1,39_ = 198.21	0.03

*^∗∗^p < 0.001. FAS, father’s affective state; IC, interactive conflict; FR, food refusal; DAS, dyad affective state.*

For the present study, inter-rater agreement between the two coders (who were specifically trained psychologists who were blind to group status) was good (Pearson *r* values, 0.8–0.9).

### Longitudinal Evaluation of Mothers’ and Fathers’ Psychopathological Risk Profiles by Group

An ANOVA of the SCL-90-R GSI scores for mothers across groups and time points revealed a significant main effect of time point (*p* < 0.01) and a significant time point × group interaction (*p* < 0.001). Scheffè’s *post hoc* tests showed that mothers in both of the treatment groups, TxMF (I–J = 0.3836; *p* < 0.05) and TxM (I–J = -0.7824; *p* < 0.001), had higher GSI scores than mothers in the Con group across both time points (**Table 4**). Importantly, the GSI score for the TxMF became significantly lower (i.e., less maladaptive) than for the TxM group at Time 2 (I–J = -0.3988; *p* < 0.05). **Table 4** shows means and η^2^ values.

**Table 4:** Mean GSI scores ± SD and η^**2**^ from the SCL-90-R for mothers and fathers by group.

Group	Parent	Mean GSI score ± SD		
		T1	T2	η^2^
TxMF	Mother	1.11 ± 0.63	0.46 ± 0.25^∗^	0.61
	Father	1.03 ± 0.55	0.48 ± 0.25^∗^	0.36
TxM	Mother	1.15 ± 0.61	1.21 ± 0.59	0.06
	Father	1.07 ± 0.60	1.23 ± 0.59	0.04
Con	Mother	0.37 ± 0.27	0.43 ± 0.26	0.03
	Father	0.34 ± 0.19	0.43 ± 0.25	0.05

*Cut-off for psychopathological risk in Italian population is ≥1 for men and women (Prunas et al., 2012). ^∗^p < 0.05.*

Likewise, an ANOVA of the SCL-90-R GSI scores for fathers across groups and time points revealed a significant main effect of time point (*p* < 0.05) and a significant time point × group interaction (*p* < 0.001). Scheffè’s *post hoc* tests showed that the mean GSI scores for the TxMF group (I–J = 0.3715; *p* < 0.05) and for the TxM group (I–J = -0.7631; *p* < 0.001) were higher than those for the Con group across both time points. Similar to our observations with the mothers’ GSI scores, only fathers in the TxMF group showed a decrease (i.e., less maladaptive) in GSI score from T1 to T2 (I–J = -0.3916; *p* < 0.05) (**Table 4**).

### Longitudinal Evaluation of Infants’ Temperamental Characteristics by Group

There was strong agreement (Pearson’s *r* = 0.87) between mothers and fathers regarding their infants’ temperament evaluations on the QUIT. Therefore, combined group mean scores for both parents were submitted to an ANOVA for each of the six domains of the QUIT, with group and time point as independent variables. The mean ± SD and η^2^ values obtained for each domain for each group by time point are reported in **Table 5**. We observed a main effect of time point on the following QUIT domain scores, namely *social orientation* (*p* < 0.001), *motor activity* (*p* < 0.01), *negative emotionality* (*p* < 0.05), *attention* (*p* < 0.01). Time point × group interactions were also observed for these same five domains: *social orientation* (*p* < 0.001); *motor activity* (*p* < 0.01) *negative emotionality* (*p* < 0.001) *attention* (*p* < 0.05). Scheffè’s *post hoc* tests further showed that, at T2, the TxM group had higher (i.e., more maladaptive) QUIT scores than the TxMF and Con groups in the domains of *motor activity* (TxMF: I–J = 0.37, *p* < 0.001; Con: I–J = 0.44, *p* < 0.001) and *negative emotionality* (TxMF: I–J = 0.4, *p* < 0.01; Con: I–J = 0.4, *p* < 0.01). Meanwhile, the TxM group had lower (i.e., more maladaptive) QUIT scores than the TxMF and Con groups in the domains of s*ocial orientation* (TxMF: I–J = -1.12, *p* < 0.001; Con I–J = -1.19, *p* < 0.001) and *attention* (TxMF: I–J = -0.34, *p* < 0.05; Con I–J = -0.38, *p* < 0.01).

**Table 5:** Questionari Italiani Temperamento domain mean scores ± SD and η^2^ values by time point for each study group and for a normative standard population (from Axia, 2002).

Subscale	TxMF			TxM			Con			Normative standard
	T1	T2	η^2^	T1	T2	η^2^	T1	T2	η^2^	
Motor activity	3.24 ± 0.22	2.88 ± 0.29^∗∗∗^	0.46	3.23 ± 0.25	3.25 ± 0.23	0.08	2.9 ± 0.45	2.82 ± 0.36	0.09	3.63 ±0.70
Attention capacity	2.97 ± 0.46	3.34 ± 0.36^∗^	0.65	2.93 ± 0.46	3.00 ± 0.37	0.02	3.32 ± 0.36	3.38 ± 0.36	0.07	3.75 ±0.72
Inhibition to novelty	3.40 ± 0.28	3.35 ± 0.33	0.08	3.37 ± 0.45	3.44 ± 0.22	0.04	3.48 ± 0.29	3.47 ± 0.33	0.08	2.53 ±0.83
Social orientation	2.91 ± 0.25	4.08 ± 0.26^∗∗∗^	0.57	2.87 ± 0.31	2.96 ± 0.27	0.05	4.06 ± 0.3	4.16 ± 0.27	0.04	4.07 ±0.65
Positive emotionality	3.30 ± 0.65	3.27 ± 0.37	0.06	3.39 ± 0.52	3.45 ± 0.51	0.08	3.47 ± 0.30	3.35 ± 0.30	0.06	4.44 ±0.72
Negative emotionality	3.82 ± 0.65	3.42 ± 0.38^∗∗^	0.61	3.83 ± 0.34	3.82 ± 0.35	0.05	3.35 ± 0.42	3.42 ± 0.30	0.08	1.97 ±0.61

*^∗^p < 0.05, ^∗∗^p < 0.01, ^∗∗∗^p < 0.001.*

## Discussion

The present study examined how two psychological 1-year treatment protocols, mother and father involvement versus mother involvement only, affected the quality of mother-infant and father-infant interactions in families in which the mothers had PND. Our results indicate that involvement of fathers was important for general treatment efficacy. We observed marked improvements in the TxMF group toward Con levels that did not occur in the TxM group. Our results indicate that a 1-year psychological intervention approach can reduce the severity of psychopathological risk of both parents when the treatment involves both parents, as in the TxMF group. On the contrary, treatment of the mother alone in the TxM group did not yield significant changes in psychometric scores. These findings are consistent with previous research (Goodman et al., 2011) showing that short-term intervention is only effective if the whole family nucleus is involved. Programs involving only the mother may be effective only if they are instituted for an extended period of time (Nylen et al., 2006). Our findings support prior research showing an association between a mother’s PND and the severity of father’s psychopathological symptoms. Furthermore, Dietz et al. (2009) found that fathers’ psychopathological symptoms moderated the relationship between maternal PND and infants’ behavioral problems in cases of mild to moderate maternal depression symptoms. Indeed, Jaffee et al. (2003) described the combination of maternal depression with paternal psychopathology as a “double checkmate” for children that are already at risk for maladaptation.

Our Con group was composed of women who, although not diagnosed with NPD, were apparently facing emotional difficulties in adjusting to parenthood. They exhibited normal-quality interactive patterns with their children during meals. However, their SVIA scores were 2–3 SDs from the norm, underscoring how even in the absence of clinical-level diagnosable symptoms, the transition to parenting can be challenging. Indeed, the incidence of affective disorders increases during the prenatal period for both mothers and fathers by 2 or 3 times with respect to the general population (Baldoni and Ceccarelli, 2010).

Our results point to temperamental difficulties perceived by parents in the children of mothers who have PND; this finding is aligned with prior studies demonstrating more complicated than normal temperamental characteristics in the children of depressed parents (Cutrona and Troutman, 1986; Beck, 1996; Bruder-Costello et al., 2007). Interestingly, we observed an improvement in social orientation domain scores on the QUIT for the TxMF group, but not for the TxM group. This dissociation supports the notion that maternal depression can impact the nature and quality of parent–child interactions and, by extension, the perception of child’s temperament (Murray and Cooper, 2003; Hanington et al., 2010). Furthermore, it has been suggested that depression weakens parents’ abilities to regulate their children’s emotions (Lovejoy et al., 2000). Our findings support the view that PND treatment should address both parents-infant relationships and not only the mother (Forman et al., 2007). In this way, we expect that early PND treatments for families should yield benefits for the children and their interactions with their parents.

Some authors suggest that differences in infants’ affective lives demonstrated at 3–6 months of age by way of mother-infant interaction assessments can provide an index of how the relationship is developing. It has been further suggested that such data can be combined with data describing negative affective experiences and self-regulatory behaviors to predict parent–child attachment quality at 12 months of age (Tronick et al., 1982; Cohn et al., 1991; Braungart-Rieker et al., 2001).

Many women in industrialized countries develop subclinical depression symptoms during the postnatal period, though they do not meet the DSM-5 criteria for PND (Weinberg et al., 2001; Austin et al., 2010; Cooper et al., 2010; Goodman and Tyer-Viola, 2010). The presence of depression symptoms correlates with negative consequences for children including the development of insecure attachment patterns in children, difficulties in cognitive and emotional development, and subsequent social and behavioral difficulties (Field, 1995; Grace et al., 2003; Tronick and Reck, 2009; Cerniglia et al., 2014b).

In a recent study of mothers with PND, Nanzer et al. (2012) found that short-term parent–child treatments with a psychodynamic approach seem to be particularly appropriate for the perinatal period because they are focused on parenthood and on the difficulties faced in relation to the identity change that becoming a parent brings. The authors found that by discussing and modifying distorted maternal representations, treatment can reduce the mother’s sense of guilt and depression and anxiety symptoms related to it. By concentrating on the representations connected to maternity, such treatments lead mothers to be more open to investigate their inner world during this transformative phase of life.

Our study has some limitations. Firstly, we did not obtain psychopathological profiles of the fathers through clinical interviews, as was done with the mothers. We also did not assess the children’s emotional adaptive functioning profiles nor the parents’ couple functioning in terms of relationship satisfaction. Nevertheless, there was strong agreement (Pearson’s *r* = 0.87) between mothers and fathers regarding their infants’ temperament evaluations on the QUIT, which is at least an indirect sign of no significant marital conflict between parents. Finally, the sample homogeneity in terms of race and geographical origin does not enable wide generalizations of the results to the general population to be made.

## Conclusion

The aforementioned limitations notwithstanding, this work adds knowledge to the PND literature by way of demonstrating that the specific short-term psychological treatment involving both parents (which has been considered in the present study) is more effective than treatment involving the mother alone. Additionally, this work provides information about the characteristics of mother-infant and father-infant dyadic interactions in families with a mother diagnosed with PND, in contrast to most previous studies that focused on assessing the stability or changes in psychopathological symptoms.

## Conflict of Interest Statement

The authors declare that the research was conducted in the absence of any commercial or financial relationships that could be construed as a potential conflict of interest.
